# Supplementary material for: Dense and accurate whole-chromosome haplotyping of individual genomes
Source: Nat Commun. 2017 Nov 3;8:1293. doi: 10.1038/s41467-017-01389-4 (PMC5670131; doi:10.1038/s41467-017-01389-4)
Supplement: Supplementary file 1 — Supplementary Information [file 41467_2017_1389_MOESM1_ESM.pdf]

Supplementary Figures

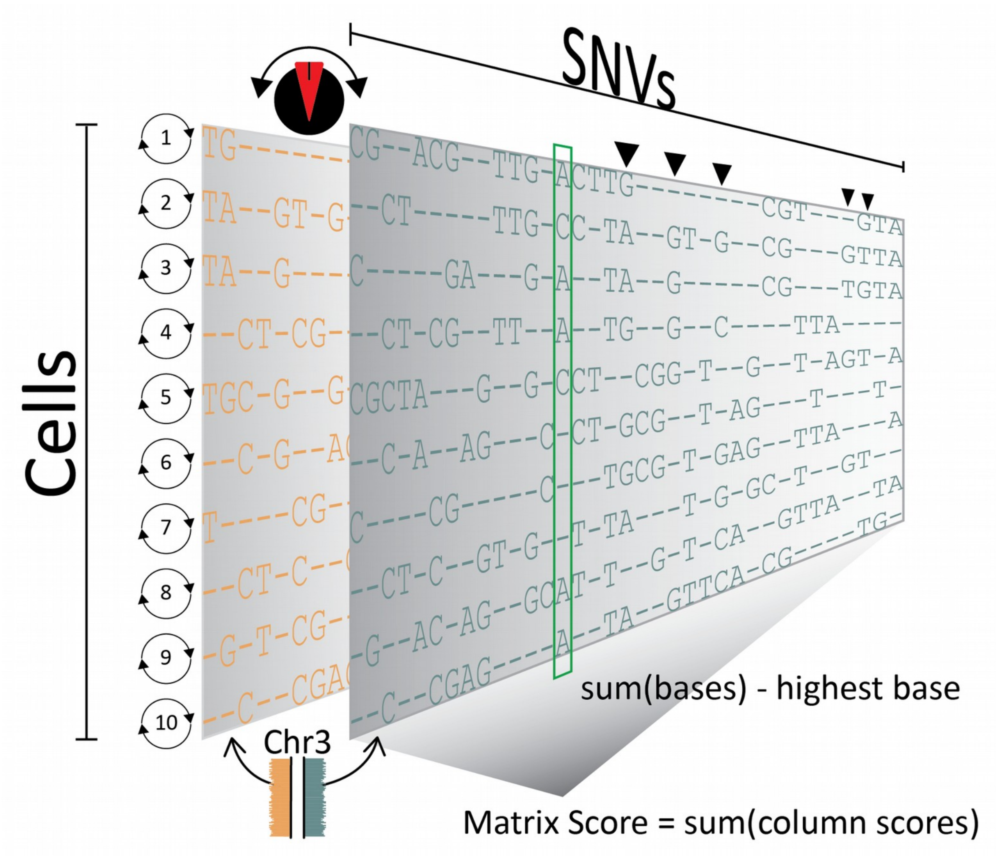

Supplementary Fig. 1: StrandPhaseR phasing algorithm.

Two parallel matrices are shown. Rows represent single cell Strand-seq libraries and columns represent variants covered in those cells. Initially, one matrix stores all alleles (SNVs) covered by Watson reads and the second matrix stores all alleles covered by Crick reads (e.g. of Chromosome 3 - Chr3) separately for every single cell. Switch button at the top of the figure illustrates swap of alleles in every row between two matrices. Matrix score is calculated for each iteration to minimize the level of disagreement seen across the columns and determine the consensus haplotype.

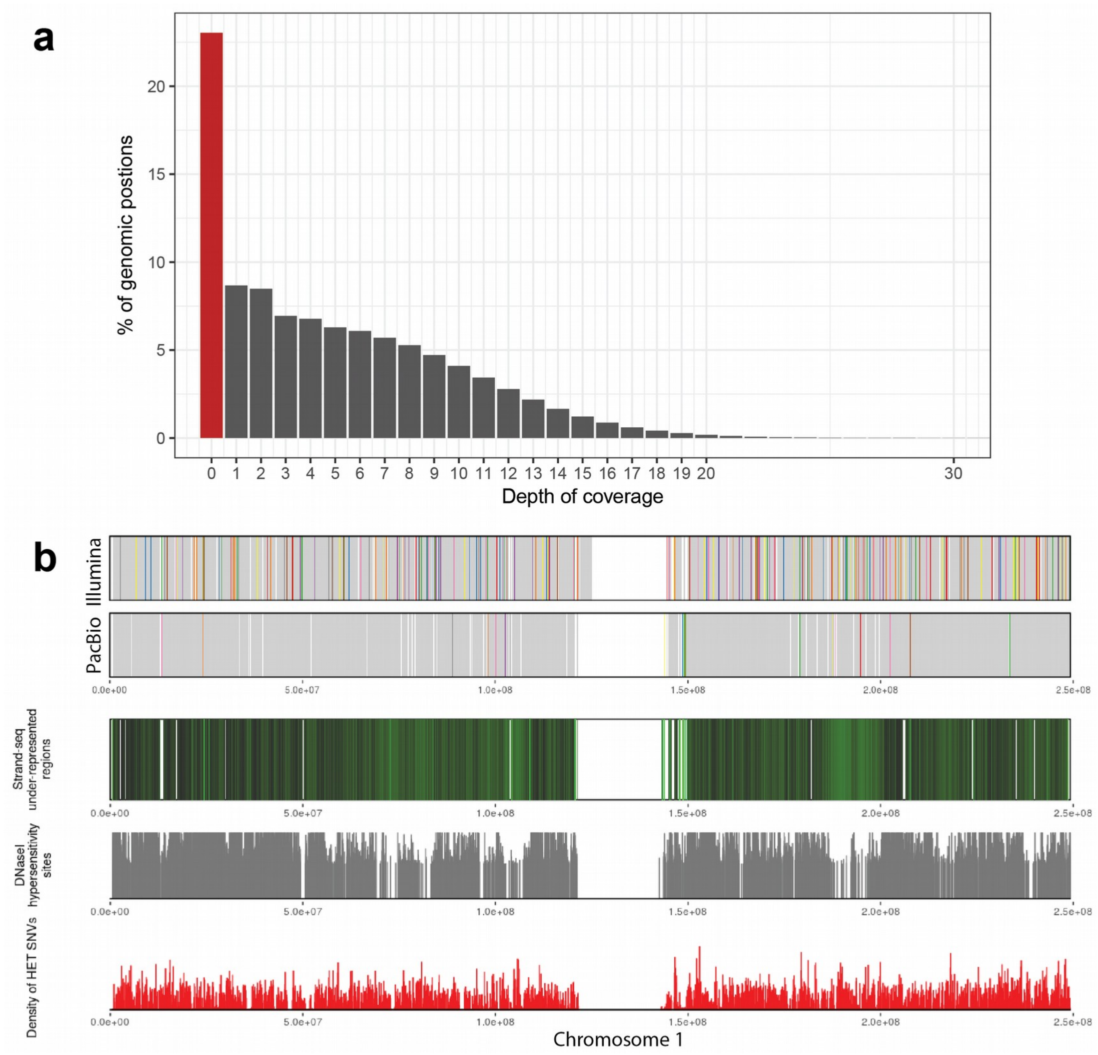

Supplementary Fig. 2: Underrepresented genomic regions in Strand-seq libraries.

a To assess the distribution of coverage in merged Strand-seq libraries (N = 134). Libraries were merged using SAMtools 'merge' function, reads were filtered for mapping quality 10, and the coverage of the genome was examined using SAMtools 'mpileup' function. On the x axis of the bar plot the depth of coverage is shown, with the corresponding percentage of the genome covered at the given depth, shown on the y-axis. The red bar highlights the portion of the genome (~ 23%) that was never covered in any Strand-seq library. The uncovered fraction may be caused by problems in assigning high-quality read alignment to the reference assembly, as well as by the inaccessibility of certain parts of the genome during library preparation. b Even at high numbers of Strand-seq libraries (N = 134) there are a number of genomic regions that could not be stitched together into a single haplotype. The black and green density track shows the genomic regions that are represented in the merged Strand-seq data for chromosome 1, where bright green highlights the underrepresented regions. We found the low density linkage information in Strand-seq data correlated more with the DNaseI hypersensitivity sites reported for NA12878 (gray track; obtained from UCSC genome browser) than with the corresponding SNV density (red track). This suggests the regions underrepresented in Strand-seq data may be due to chromatin organisation of the genome (as the Strand-seq protocol utilizes an MNase-digestion fragmentation step).

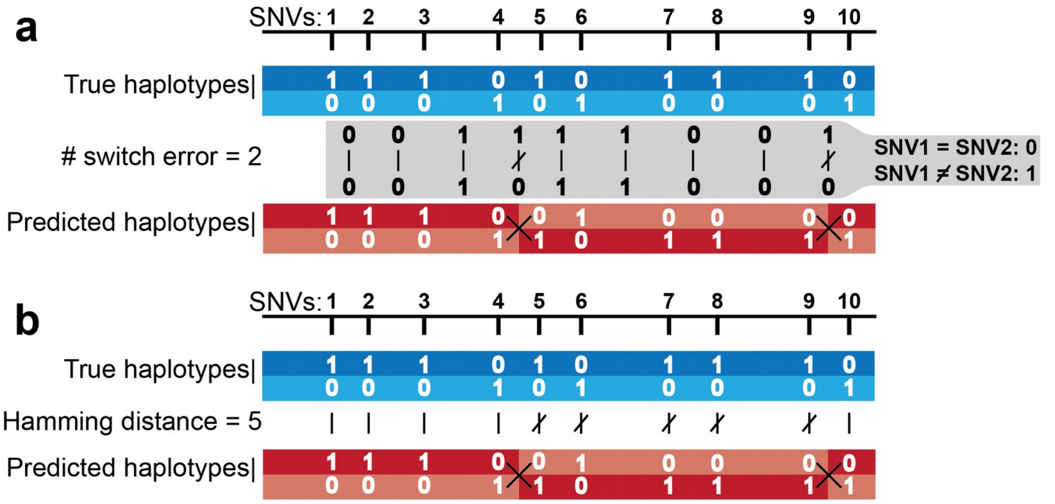

Supplementary Fig. 3: Quality measures used to evaluate predicted haplotypes.

Hypothetical phasing of 10 single nucleotide variants (SNVs) along a defined chromosomal region is shown here. Each heterozygous SNV is represented in its two allelic forms (0 – reference allele, 1 – alternative allele). True (reference) haplotypes are distinguished in blue colors and predicted haplotypes in red. a To count the number of switch errors (black crosses) between the true and predicted haplotypes, neighbouring pairs of SNVs are compared along each haplotype and recorded as a new binary string of 0's and 1's depending on whether the allele state changes (see gray box). A zero value is assigned if the given pair of SNVs have the same value, otherwise a value of 1 is assigned value 1. The absolute number of differences in the binary string generated for the true and predicted haplotypes is reported as the total number of switch errors. b To calculate the Hamming distance, the absolute number of differences between reference and predicted haplotypes is calculated for all SNV positions. In addition we calculate block-wise Hamming distance which represents a cumulative sum of all Hamming distances across all phased segments (see **Supplementary Fig. 5**).

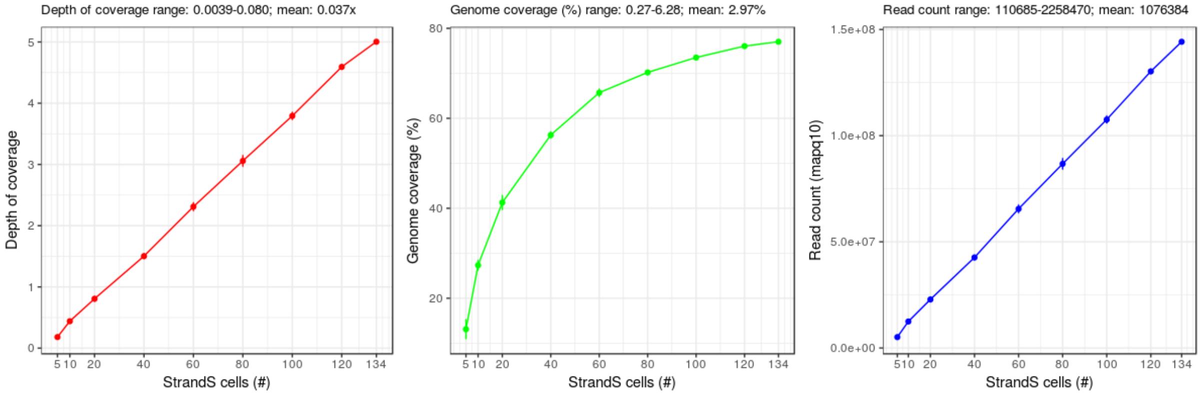

Supplementary Fig. 4: Coverage summary for various numbers of Strand-seq libraries

Plots shows the depth of coverage, genome coverage and number of reads when using different numbers of Strand-seq libraries (x axis). We have performed 5 randomized selections of any given library count, reflected in the error bars. Depth of coverage is calculated as an overall number of bases sequenced per genomic position (excluding gaps ("N") in the genome). Genome coverage is calculated as a percentage of genomic positions (excluding gaps in the genome) covered with at least one read. Duplicate reads and reads with mapping quality < 10 were filtered out prior to coverage analysis.

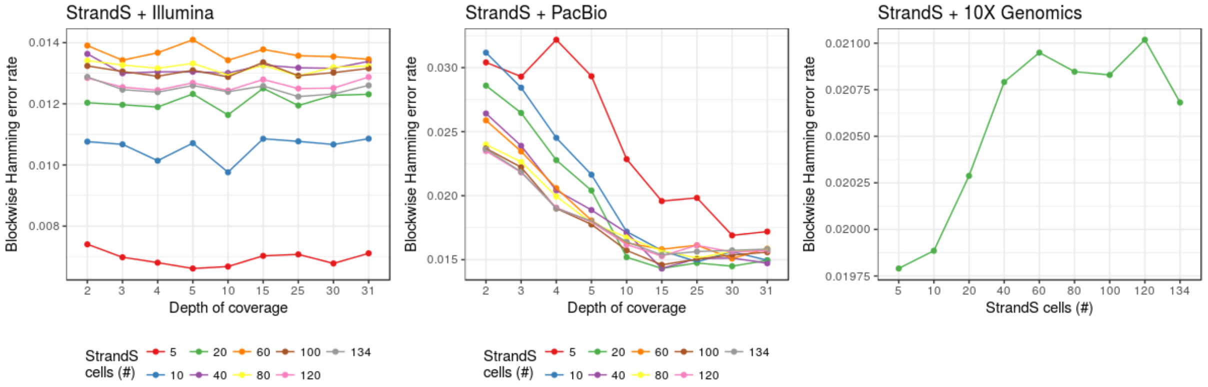

Supplementary Fig. 5: Comparison of block-wise Hamming distances

Each sequencing technology is combined with various numbers of Strand-seq cells and the block-wise Hamming error rate is calculated for each combination as the sum of all Hamming distances across all phased haplotype segments divided by the total length of these segments.

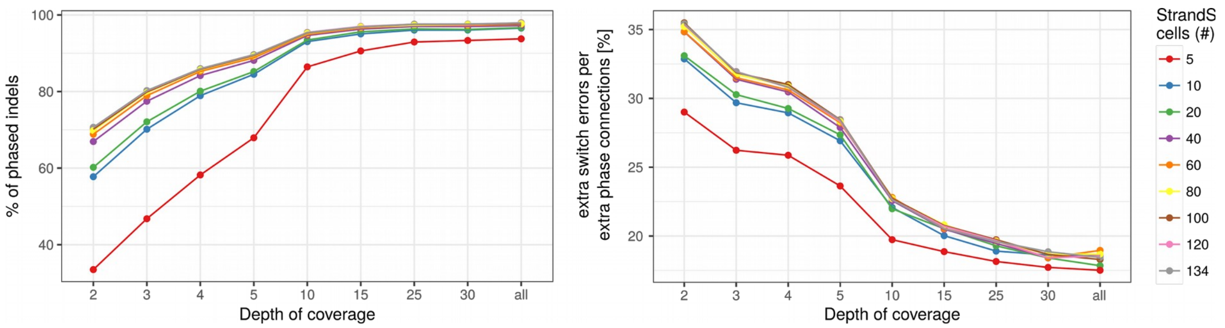

Supplementary Fig. 6: Indel phasing performance when combining Strand-seq and PacBio.

Plot shows the performance of indel phasing on Chromosome 1 when combining various numbers of Strand-seq cells (5, 10, 20, 40, 60, 80, 100, 120, 134) with selected coverage depths of PacBio sequencing data (2, 3, 4, 5, 10, 15, 25, 30, >30-fold). Left: percentage of indels phased as part of the largest block. Right: extra switch errors per extra phase connections (in the largest block), that is, we count the number of additional switch errors compared to when only phasing SNVs and divide by the number of extra phase connections (the difference of phase connections in the largest block when only phasing SNVs compared to phasing SNVs and indels), where "phase connection" is defined to be a pair of phased heterozyous variants consecutive in their phased block (the number of phase connections in the largest segment is hence equal to the number of heterozygous variants in the largest segment minus one).

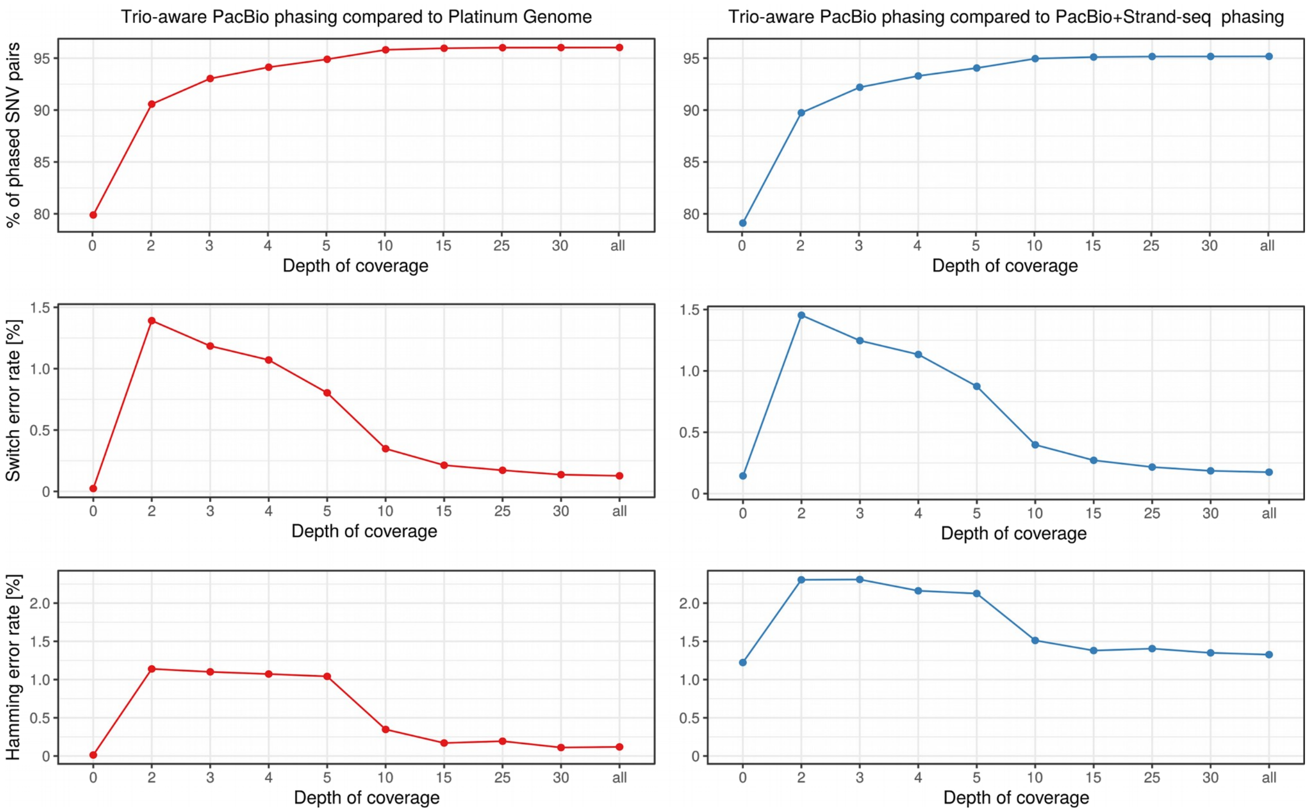

Supplementary Fig. 7: Evaluation of trio-aware read-based phasing.

Genotype data of parents (NA12891 and NA12892) and child (NA12878) were used in conjunction with PacBio reads for the child at the designed coverage (x-axes) to obtain a phasing. Plots in the left column (red) compare the resulting phasings to the Platinum genomes, plots in the right column (blue) compare the resulting phasings to the combination of 134 Strand-seq cell and full PacBio coverage (in for a single individual). The plots show the fraction of phased heterozygous SNV pairs (top), the switch error rate (middle), and the Hamming error rate (bottom).

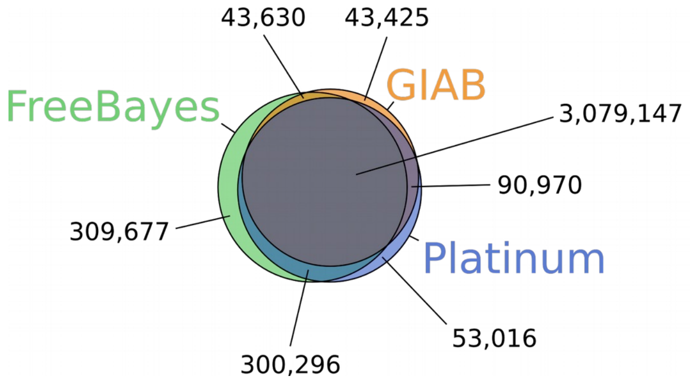

Platinum	GIAB	FreeBayes	Count
0/1	0/1	0/1	1,872,568
0/1	0/1	1/1	171
0/1	1/1	0/1	1
0/1	1/1	1/1	3
1/1	0/1	1/1	1
1/1	1/1	0/1	377
1/1	1/1	1/1	1,206,026

Supplementary Fig. 8: Concordance of different SNV sets.

A Venn diagram of bi-allelic SNV calls (heterozygous or homozygous) in the three call sets (FreeBayes, GIAB, Platinum) is shown on the left. For the calls common to all thee callers, a genotype confusion matrix is shown on the right.

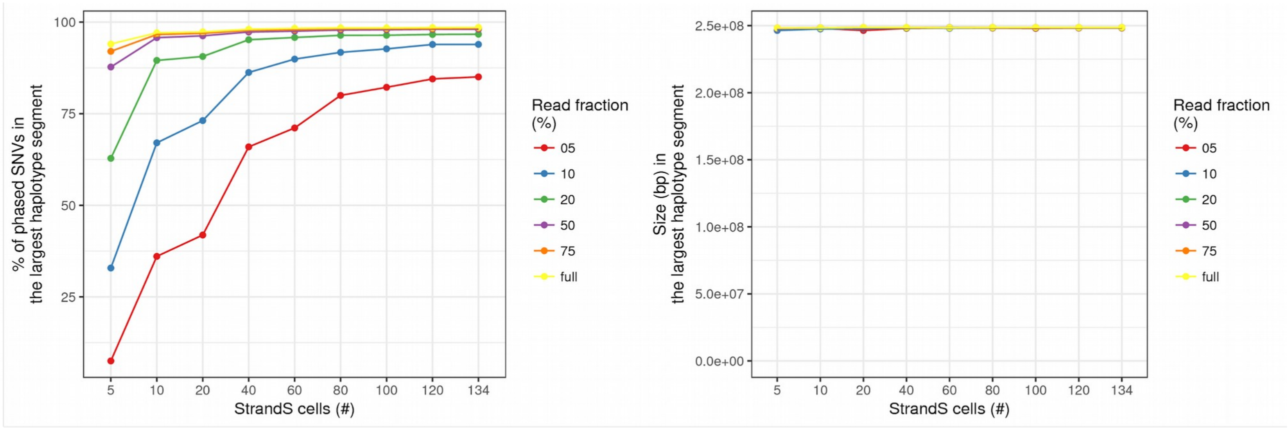

Supplementary Fig. 9: Effect of downsampling Strand-seq libraries.

To test the scaffolding capabilities of Strand-seq libraries even with lower coverages than present in libraries used in this paper, we have downsampled aligned Strand-seq reads on Chromosome 1 to various fractions of the original number of reads (5%, 10%, 20%, 50%, 75%). We have run StrandPhaseR on the reduced libraries (for different number of Strand-seq cells: 5, 10, 20, 40, 60, 80, 100, 120, 134) and combined the resulting sparse haplotypes with the full coverage PacBio data. **a** Shows the percentage of phased SNVs in the largest haplotype segment. **b** Shows the span of the largest phased haplotype segment.

Supplementary Tables

Chromosome1	Illumina data	PacBio data	10X Genomics
AVG length of mapped DNA fragments	248 bp	4,429 bp	139 bp
AVG depth of coverage	41.06x	45.78x	25.3x

Supplementary Table 1: Summary measures for PacBio and Illumina data using Chromosome 1 as an example.

# Strand-seq libaries	134
Sequencing protocol	Paired-end
Read length	100bp
AVG genome coverage per library	2.97%
AVG depth of coverage per library	0.037
Genome coverage in merged libraries	77.02%
Depth of coverage in merged libraries	5.004
Depth of coverage in all WC regions	2.641

Supplementary Table 2: Summary measures for Strand-seq libraries.

Genome coverage is calculated as a percentage of genomic positions (excluding gaps in the genome) covered with at least one read. Depth of coverage is calculated as an overall number of bases sequenced per genomic position (excluding gaps in the genome). Coverage was calculated after filtering duplicate reads and reads with mapping quality < 10.

	Covered variants (%)	Switch error (%)	Hamming error (%)
~30-fold Illumina + 40 StrandS 1.5-fold	68.1	0.45	0.99
~10-fold PacBio + 10 StrandS 0.44-fold	95.56	0.25	0.91
~25-fold 10xGen + 10 StrandS 0.44-fold	98.13	0.05	2.18

Supplementary Table 3: Summary of integrative whole-genome phasing for recommended technology combinations.

	Covered variants (%)	Switch error (%)	Hamming error (%)
~30-fold Illumina + 40 StrandS 1.5-fold	66.2	0.51	1.23
~10-fold PacBio + 10 StrandS 0.44-fold	94.4	0.34	1.14
~25-fold 10xGen + 10 StrandS 0.44-fold	97.8	0.06	1.81

Supplementary Table 4: Summary of integrative whole-genome phasing for recommended technology combinations when using the FreeBayes SNV call set.

Supplementary Notes

Supplementary Note 1: Comparison of SNV call sets and their influence on phasing results

Our phasing pipeline works on an input set of variants, whose quality can affect the results. In this section, we collect some statistics on the concordance of different SNV call sets. First, we additionally obtained a Genome in a Bottle (GIAB) call set (release v3.3.2)[1] and compared it to the Platinum Genomes calls in use, both for GRCh37. These two sets share 3,170,117 bi-allelic SNVs, while 353,312 are only present in the Platinum Genomes and 87,055 are only present in the GIAB set. For the intersection, genotypes agree for 3,170,110 out of 3,170,117 SNVs (99.9998%).

Both the GIAB and the Platinum Genomes call sets are highly curated and data of this quality might not be always available in application scenarios. We therefore downloaded the BAM file of aligned reads for NA12878 (from the Platinum Genomes) and ran FreeBayes (v1.0.2) with standard parameters on these data, which resembles a standard workflow. The resulting variants were filtered for quality values of at least 30. The resulting call set shows very good agreement to the GIAB and Platinum call sets (Supplementary Figure S8).

To verify that a standard SNV call set yields comparable phasing results, we ran the same phasing pipeline for the three recommended technology combinations on the FreeBayes SNV set. The results are shown Supplementary Table S4. Comparing these results to Supplementary Table S3 (which contains the corresponding results when starting from the Platinum Genomes SNV set), we observe slightly worse (yet comparable) results. When combining PacBio (10-fold) with Strand-seq data (10 cells), for instance, the switch error rate changes from 0.25% to 0.34% and the Hamming error rate changes from 0.91% to 1.14%.

1 ftp://ftp-trace.ncbi.nlm.nih.gov/giab/ftp/release/NA12878_HG001/NISTv3.3.2/GRCh37/HG001_GRCh37_GIAB_highconf_CG-IllFB-IllGATKHC-Ion-10X-SOLID_CHROM1-X_v.3.3.2_highconf_PGandRTGphasetransfer.vcf.gz

Supplementary Note 2: WhatsHap command lines and workflow

We provide a Snakemake workflow to reproduce our complete analysis at (and also include a tarball as Supplementary Data). Although also included in the Snakemake workflow, we here list how to invoke WhatsHap for the reader's convenience.

Phasing

After Strand-seq data has been processed by StrandPhaseR into VCFs that contain haplotype scaffolds, we run WhatsHap to combine PacBio or Illumina reads with StrandSeq data:

```
whatshap phase [--indels] --distrust-genotypes --sample
NA12878 --reference <hg19.fasta> <platinum-unphased.vcf>
<strandseq.vcf> <(illumina|pacbio).bam> --output
<output.vcf>
```

Note that we ran the above command without `--indels` to obtain the results mentioned in the main text and present some results obtained with indels in Supplementary Figure 6. To combine Strand-seq data with pre-phased 10X Genomics haplotype segments (from LongRanger), we use the following command line:

```
whatshap phase [--indels] --distrust-genotypes --sample
NA12878 <platinum-unphased.vcf> <strandseq.vcf> <10X-
phasing.vcf> --output <output.vcf>
```

Comparison

We also use WhatsHap to compare the obtained phasings to the ground truth provided by the Platinum genomes:

```
whatshap compare --names benchmark,whatshap --tsv-pairwise
{output} --only-snvs <platinum.vcf> <whatshap-phased.vcf>
```

Supplementary Note 3: Trio-aware read-based phasing

To perform trio-aware read-based phasing, we have used genotype data for parents (NA12891 NA12892) and child (NA12878) in conjunction with PacBio reads from the child to perform phasing in the PedMEC model (Minimum Error Correction on Pedigrees)[1]. To obtain genotypes for all three family members, we ran FreeBayes (v1.0.2) on all three samples[2] to re-genotype all SNVs reported for NA12878 in the Platinum genomes data set (using options "--haplotype-basis-alleles" and "-@"). The resulting genotypes were filtered for those with a quality of 30 or above. We then used WhatsHap in pedigree mode (option --ped) providing it with family genotypes and different coverages of PacBio reads for the child (0x, 2x, 3x, 4x, 5x, 10x, 15x, 30x, all). Note that coverage 0x corresponds to pure genetic haplotyping relying solely on the genotypes. Pure genetic haplotyping can only phase variants that are not heterozygous in all individuals (i.e. homozygous in at least one), which lead to 83.0% of all variants being phased. **Supplementary Fig. 7** shows a comparison of the obtained haplotypes to the Platinum Genomes phasing (left, red) and the haplotypes resulting from combining Strand-seq (all 134 cells) with PacBio (full coverage) in single individual mode (as discussed in the main text). By increasing the PacBio coverage from 0x to 10x, we are able to increase the completeness from 83% to 96% phased heterozygous SNVs. Switch and Hamming error rates indicate excellent agreement with the Platinum Genomes phasing (**Supplementary Fig. 7**, left). Since the genotype data we use for PedMEC phaing rely on the Platinum Genome BAM files, we aimed to provide additional evidence for the quality of the results. To this end, we note that the comparison to the haplotypes obtained from Strand-seq and PacBio (without using family information) also indicates very good agreement (**Supplementary Fig. 7**, right).

2 BAM files downloaded from ftp://ftp.sra.ebi.ac.uk/vol1/ERA172/ERA172924/bam/<sample>_S1.bam.

Supplementary Note 4: Phasing Indels

In the main text, we only considers SNVs, although our pipeline can also handle indels. We reran our pipeline for jointly phasing SNVs and indels using the combination of Strand-seq and PacBio data. The results are displayed in **Supplementary Fig. 6**. While we are able to include a high number of indels in the largest segment (93.0% when using 10 Strand-seq cells and 10-fold PacBio coverage, 97.9% when using 134 Strand-seq cells and full PacBio coverage), the observed error rates are much higher than for SNVs (22.1% when using 10 Strand-seq cells and 10-fold PacBio coverage, 18.4% when using 134 Strand-seq cells and full PacBio coverage). These error rates are consistent with earlier findings[2]. We attribute the difference in performance between SNVs and indels to low complexity indels (such as STRs).

References:

1. Garg, S., Martin, M., Marschall, T. Read-based phasing of related individuals. *Bioinformatics* **32**(12), 234–242 (2016).
2. Martin, M., Patterson, M., Garg, S., Fischer, S.O., Pisanti, N., Klau, G.W., Schönhuth, A., Marschall, T. WhatsHap: fast and accurate read-based phasing, bioRxiv, doi:10.1101/085050, 2016.
